# The structure and function of rhizosphere bacterial communities: impact of chemical vs. bio-organic fertilizers on root disease, quality, and yield of *Codonopsis pilosula*

**DOI:** 10.3389/fmicb.2024.1484727

**Published:** 2024-10-21

**Authors:** Bin Huang, Yuxuan Chen, Yi Cao, Dongyang Liu, Hua Fang, Changchun Zhou, Dong Wang, Jie Wang

**Affiliations:** ^1^Pest Integrated Management Key Laboratory of China Tobacco, Tobacco Research Institute of Chinese Academy of Agricultural Sciences, Qingdao, China; ^2^Guizhou Academy of Tobacco Science, Guiyang, China; ^3^Institute and Enterprise Joint Creation of Tobacco Technology Center, Sichuan Provincial Tobacco Company Liangshanzhou Company, Liangshanzhou, China; ^4^Shandong Hezhong Kangyuan Biotechnology Co., Ltd, Zibo, Shandong, China; ^5^Department of Vector Biology and Contro, Jinan Center for Disease Control and Prevention, Jinan, China

**Keywords:** chemical and bio-organic fertilizers, *Codonopsis pilosula*, co-occurrence network, microbial community, yield and quality

## Abstract

**Introduction:**

Long-term use of chemical fertilizers (CFs) can cause soil compaction and acidification. In recent years, bio-organic fertilizers (BOFs) have begun to replace CFs in some vegetables and cash crops, but the application of CFs or BOFs has resulted in crop quality and disease occurrence.

**Methods:**

This study aimed to analyze the microbial mechanism of differences between CFs and BOFs in root disease, quality, and yield of tuber Chinese herbal medicine. We studied the effects of CFs, organic fertilizers, commercial BOFs, biocontrol bacteria BOFs, and biocontrol fungi BOFs on rhizosphere microbial community structure and function, root rot, quality, and yield of *Codonopsis pilosula* at different periods after application and analyzed the correlation.

**Results and discussion:**

Compared to CFs, the emergence rate and yield in BOF treatments were increased by 21.12 and 33.65%, respectively, and the ash content, water content, and disease index in the BOF treatments were decreased by 17.87, 8.19, and 76.60%, respectively. The structural equation model showed that CFs promoted the quality and yield of *C. pilosula* by influencing soil environmental factors, while BOFs directly drove soil bacterial community to reduce disease index and improve the quality and yield of *C. pilosula*. There was a stronger interaction and stability of soil microbial networks after BOF treatments. *Microlunatus*, *Rubrobacter*, *Luteitalea*, *Nakamurella*, and *Pedomicrobium* were identified as effector bacteria, which were related to disease prevention and yield and quality increase of *C. pilosula*. Microbial functional analysis indicated that the signal transduction and amino acid metabolism of soil bacteria might play a major role in improving the quality and yield of *C. pilosula* in the early and middle growth stages. In conclusion, compared to CFs, BOFs obtained a lower disease index of root rot and a higher quality and yield of *C. pilosula* by changing the structure and function of the rhizosphere bacterial community.

## 1 Introduction

*Codonopsis pilosula* is a perennial herbaceous plant and an important Chinese herbal medicine. Its fleshy roots are widely used in the field of medicine, and they can be used as both medicine and food (Lodi et al., 2023). Perennial continuous cropping can lead to the accumulation of pathogenic bacteria, and this can result in the severe root rot of *C. pilosula*. One of the main goals of current research in the production of Chinese herbal medicines, including *C. pilosula*, is to develop approaches for enhancing yield and quality and the control of plant diseases without compromising soil health.

Bio-organic fertilizers (BOF) are environmentally friendly fertilizers that can be used for the control of pest populations and for enhancing soil properties. Some of the advantages of BOFs include their positive effects on soil microbial activities and their ability to enhance soil properties and prevent soil-borne diseases, as well as some of the problems caused by *CF* application, including soil compaction and acidification and ecological imbalances (Rani et al., 2021). BOF application has been shown to be effective in enhancing the production of agricultural crops. Wang et al. (2022a) reported that *Bacillus subtilis* BOF can promote cabbage growth, with plant height and biomass being 1.20 and 1.93 times greater in the BOF group than in the *CF* group on the 30th day of the experiment. The sugar–acid ratio of apples was greater under BOF application than under organic fertilizer (OF) application, and this is associated with differences in sucrose accumulation and citric acid degradation (Wang et al., 2022b). The efficacy of different crop protection practices has been studied for several crops, including tobacco, bananas, and tomatoes. BOFs have been shown to be more effective than CFs or OFs in minimizing the deleterious effects of tobacco black shank (Chen et al., 2023), banana wilt (Tao et al., 2020), and tomato bacterial wilt (Shen et al., 2021). However, compared to CFs, the mechanism by which BOFs enhances the yield and quality of root herbs at the microbiological level has not yet been elucidated.

Soil microbial structure and function are likely key for mediating differences in the positive effects of BOFs and CFs on the quality and yield of crops or Chinese herbs. The application of CFs is considered an important source of crop nutrient elements, including macroelements and trace elements, which play key roles in diverse processes, including protein synthesis (Khan et al., 2021), energy conversion processes (Wang et al., 2017), water regulation (Hartmann et al., 2021), and regulation of enzyme activity and hormone synthesis (Li et al., 2021). The application of BOFs has various effects on soil microorganisms, including their ability to respond to abiotic and biotic stresses. BOFs have thus received a lot of research attention. BOF application after fumigation has been shown to increase soil pH and the abundance of 13 functional genes related to amino acid metabolism, suggesting that soil ecological health was improved following the application of BOF (Huang et al., 2023). The BOF SQR9 has been shown to increase pear yield by enhancing the abundance and functional diversity of the rhizosphere microbiome. Specifically, the abundance and functional diversity of the rhizosphere were 21 and 8% higher following BOF application compared to *CF* and OF application, respectively (Wang et al., 2023). BOFs have been shown to have indirect and lagged effects (Chen et al., 2023), with fruit quality improving more significantly following BOF application than the application of traditional OFs (Wang et al., 2022a; Wang et al., 2022b; Wang et al., 2022c). This improvement may stem from the diverse functions of soil microorganisms.

In this study, the roots of *C. pilosula*, a Chinese herbal medicine, were explored by conducting an experiment in which plants were subjected to treatments with CFs, OFs, and different BOFs. We (1) measured the emergence rate, incidence rate, yield, and quality of *C. pilosula*; (2) determined soil physicochemical properties at different periods, *α* and *β* diversity of soil microorganisms; (3) analyzed the factors leading to the differences between CFs and BOFs using a structural equation model (SEM); (4) identified differential microorganisms and core microorganisms in the rhizosphere soil through network analysis as key microorganisms affecting *C. pilosula*; and (5) employed ternary analysis, correlation analysis, and the Mantel test of microbial function to reveal the potential mechanisms by which BOFs influence the quality and yield of *C. pilosula* through microbial reshaping at different times. This study provides new insights into using different fertilizers to improve the quality and yield of tuber Chinese herbal medicine, highlighting the feasibility of BOFs as an alternative to chemical fertilizers in Chinese herbal medicine cultivation.

## 2 Materials and methods

### 2.1 Field experimental design

The experimental site was in Mianliuping Village, Dingxi City, Gansu Province (35.13675°N, 104.215467°E). *C. pilosula* has been continuously cultivated at the experimental site for several years; the incidence of root rot in the field plot was 20–30%. *C. pilosula* seedlings with good growth and no disease were transplanted from the seedling nursery on 20 April 2022. Fertilizers were applied the day before *C. pilosula* transplanting. The experiment comprised five fertilization treatments: CK: 600 kg/ha of conventional chemical fertilizer (total nutrients ≥40%, N–P_2_O_5_–K_2_O = 15–15–10%); T1: 1500 kg/ha of prickly ash seed oil meal OF (without biocontrol bacteria); T2: 1500 kg/ha of commercial BOF (*Bacillus* complex); T3: 1500 kg/ha of *Trichoderma* BOF (prickly ash seed meal OF + *Trichoderma asperellum*); and T4: 1500 kg/ha of *Bacillus* BOF (prickly ash seed meal OF + *Bacillus amyloliquefaciens* + *B. subtilis*). The size of each plot was 200 m^2^, and a total of 12,000 plants of *C. pilosula* were transplanted. The plots were randomly arranged, and three replications of each treatment were performed. All plants were cultivated in an open field and cultivated per standard local practices. *C. pilosula* was harvested on 23 October 2024, 5 months after transplanting. The compound chemical fertilizers were obtained from Su Di Fertilizer Co., Ltd. in Gansu Province. The commercial BOFs were obtained from Hebei Rundong Fertilizer Co. Ltd. *Trichoderma* and *Bacillus* BOFs were provided by the Tobacco Research Institute of the Chinese Academy of Agricultural Sciences (Huang et al., 2021).

### 2.2 Determination of emergence rate, disease index, quality, and yield of *C. pilosula*

The emergence rate of *C. pilosula* in each plot was determined at the seedling stage on 25 May 2024. and *C. pilosula* were considered to have emerged if they were approximately 2 cm above the ground. The disease index of root rot of *C. pilosula* was measured at the time of harvesting. The disease index was determined using the following four-graded scale: Grade 0, disease spots are absent along the entire root system; Grade 1, the area with lesions is less than one-third of the entire area of the roots; Grade 2, the area with lesions accounts for one-third to two-thirds of the entire root area; and Grade 3, the area with lesions is greater than two-thirds of the entire root area. The formula for the disease index was based on a previous study (Carlos, 2022). During the harvest period, a total of 30\u00B0*C. pilosula* were randomly excavated from each plot, and branches and large pieces of soil were removed. Samples were dried at 50°C to maintain low moisture content. After drying, the yield of *C. pilosula* was weighed and counted for each treatment. The water content of *C. pilosula* samples was determined according to General Rule 0832 from the 2015 edition of the Pharmacopoeia of the People’s Republic of China 2015 (Part 4). The total ash content of *C. pilosula* samples was determined according to General Rule 2302 of the Pharmacopoeia of the People’s Republic of China (National Pharmacopoeia Commission, 2015). Three parallel experiments were performed. The quality of *C. pilosula* is mainly determined by its ash and water content. According to the first edition of the Pharmacopoeia of the People’s Republic of China (2015), the water content of *C. pilosula* should not exceed 16%, with approximately 10% being optimal. Additionally, the total ash content should not exceed 5%; lower total ash content indicates better quality of *C. pilosula*.

### 2.3 Collection of soil samples and determination of physicochemical properties

Soil samples were collected on the 30th, 90th, and 150th days after fertilization. The *C. pilosula* was uprooted, and most of the soil was shaken off. The soil remaining on the surface of the roots was collected with a brush, which was the rhizosphere soil. A total of three replicate soil samples were taken. The obtained soil samples were stored at −20°C for DNA extraction and physical and chemical analyses. Soil ammonium N (NH_4_^+^-N) and nitrate nitrogen (NO_3_^−^-N) were extracted using 2 M KCl and then determined using a Flow Analytical System (Yan et al., 2017). A sodium bicarbonate extraction agent was used to extract soil-available phosphorus (AP), and the molybdenum antimony resistance colorimetric method was used to measure AP (Olsen, 1954). Soil-available potassium (AK) was extracted from neutral 1 mol/L ammonium acetate solution and determined using a Model 410 flame photometer (Franz et al., 1996). The soil organic matter (OM) was determined using the potassium dichromate method (Xu et al., 2022). Soil pH and water content were measured in a 1:2.5 soil/water suspension.

### 2.4 High-throughput sequencing

An E.Z.N.A.^®^ soil DNA kit (Omega Bio-tek, Norcross, GA, USA) was used to extract the total genomic DNA of the rhizosphere soil samples per the manufacturer’s instructions. The V3–V4 variable region of the *16S rRNA* gene was sequenced using the forward primer 338F and the reverse primer 806R (Xu et al., 2016) carrying barcode sequences and the extracted DNA as a template. PCR was conducted in 20 μL reactions, and the reaction system and thermal cycling conditions referred to our previous studies (Huang et al., 2023). The samples were then preserved at 4°C. A total of three replicate reactions were performed per sample. PCR products from the same sample were mixed, and 2% agarose gel electrophoresis was performed to recover the samples. The products were purified, recovered, detected, and quantified, and then the PCR library was constructed. Illumina’s MiSeq PE300/NovaSeq PE250 platform (Shanghai Meiji Biomedical Technology Co., Ltd.) was used to sequence the libraries.

### 2.5 Bioinformation and statistical analysis

FLASH software (v1.2.11) was used to splice the sequences derived from the offline raw data. The QIIME (v1.9.1) platform was used for quality control of the original sequences, and the UCHIME algorithm was used to detect and remove chimeric sequences. The Uparse (v11) pipeline was used to cluster operational taxonomic units (OTUs) with at least 97% similarity (Adams et al., 2013). The taxonomic identity of the bacterial and fungal sequences was determined using the SILVA (v138) database for bacteria with the Mothur algorithm and the UNITE (v8.0) database for fungi (Edgar et al., 2011). The abundance of OTUs was standardized relative to the least abundant sequence in the samples; diversity analysis was conducted using standardized OTU abundance data. The *α*-diversity of the Simpson and Chao1 indices of microbial communities was analyzed using Mothur (v.1.30.0) software (Schloss et al., 2009). Principal coordinate analysis (PCoA) was performed using Bray–Curtis distances. The relative abundances of species were analyzed using Python (v.2.7.0).

The pheatmap package and vegan package in R were used to generate heatmaps and determine statistical correlations. SEM in IBM SPSS Amos 26 was used to evaluate the effects of soil environmental factors and microorganisms on the quality and yield of *C. pilosula* (Chu et al., 2022). Environmental factors with significant effects on soil microbial communities were determined using the Mantel tests (Li et al., 2022). Visual maps for correlation analysis of environmental factors (Pheatmap), Ternary maps for three temporal soil microbial functions (Ternary), and pie maps for contribution of species functions (vegan) were drawn using R (4.3.2). The soil microbial co-occurrence network was constructed using the Hmisc package and visualized with Gephi (0.10.0). PICRUSt was used to predict the microbial function of bacterial *16S rRNA* sequencing data (Huang et al., 2021). After OTUs standardization, the KEGG database annotation was performed to analyze microbial metabolic pathways. The significance of differences in emergence rate, disease index, quality, yield, and soil physicochemical properties was determined using a one-way analysis of variance and Duncan’s multiple range test.

## 3 Results and discussion

### 3.1 Effects of each treatment on the emergence rate, disease index, quality, and yield of *C. pilosula*

**Figure 1:**
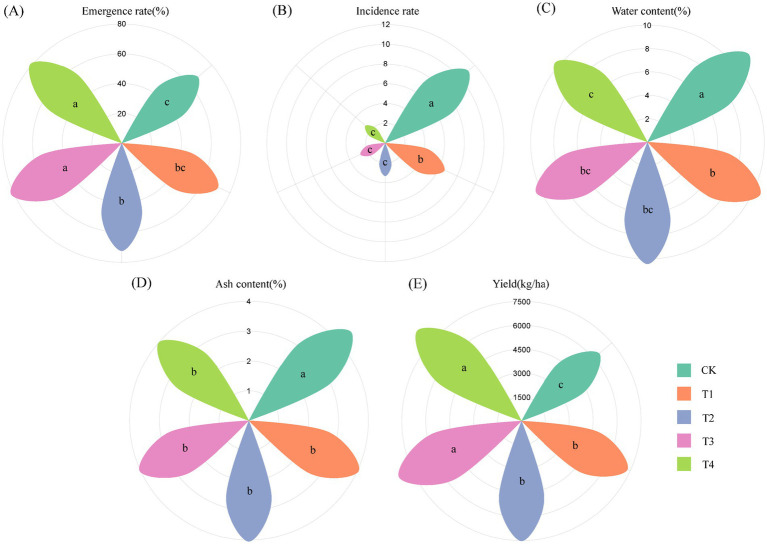
Emergence rate, disease index, yield, and quality of *C. pilosula* following treatment with *CF*, OF, and BOF. (A) Emergence rate of *C. pilosula*; (B) incidence of root rot; (C) moisture content of *C. pilosula*; (D) ash content of *C. pilosula*; (E) yield of *C. pilosula*. Specific information on each indicator is provided in Supplementary Table S1. Different letters (a, b, c) indicate significant differences between treatments.

The emergence rate was the lowest in the chemical fertilizer (CK, 67.62%) and the highest in T3 (81.90%). The emergence rate was significantly higher in T2, T3, and T4 than in the CK (Figure 1A). The exogenous application of BOFs has been shown to promote the metabolic activities of soil microorganisms, including respiration, and the water generated might play a key role in inducing the emergence of *C. pilosula* (Carlos, 2022; Zhao et al., 2019). The disease index was the highest in the CK (11.11) and the lowest in T4 (2.60). The disease index was significantly lower in T1, T2, T3, and T4 than in the CK, and the disease index was significantly lower in T2, T3, and T4 than in T1 (Figure 1B), suggesting that BOFs could effectively control the root rot of *C. pilosula*.

The water content was significantly higher in the CK (11.23%) than in the other four treatments, and the water content was the lowest in T4 (10.31%). The ash content was significantly higher in the CK (4.87%) than in the other four treatments, and the total ash content was the lowest in T4 (4.00%). According to the first edition of the Pharmacopoeia of the People’s Republic of China (2020), the water content of *C. pilosula* should not exceed 16%, and the total ash content should not exceed 5%. A previous study has shown that severe worm infestations can occur when the water content of *C. pilosula* is >15%, and worm infestations can be prevented when the water content is <13% (Leng et al., 2023). The quality and ash content of *C. pilosula* are inversely related. The fresh root yield was significantly higher in T3 and T4 than in the other three treatments (Figures 1C–E; Supplementary Table S1).

### 3.2 Effects of fertilizations on the soil physicochemical properties

At 90 days after treatment, the NH_4_^+^-N was significantly higher in T1, T2, and T3 than in the CK. The soil NO_3_^−^-N content was significantly higher in T4 than in the CK at 30 days and 90 days after treatment. BOF application has been shown to promote the N fixation of microbes in soil and reduce the loss of N (Rahimi et al., 2021) (Table 1).

**Table 1:** The physicochemical properties of soil in the chemical fertilizer, OF, and BOF treatments.

Sampling days	Treatment	NH_4_^+^-N (mg/kg)	NO_3_^−^-N (mg/kg)	Available P (mg/kg)	Available K (mg/kg)	SOM (mg/kg)	pH (1:2.5)	EC (μs/cm)
Day 30	CK	35.07 ± 1.39a	17.07 ± 0.38c	117.97 ± 3.83a	238.37 ± 6.93a	10.77 ± 0.30d	7.06 ± 0.01a	130.13 ± 2.05b
	T1	36.60 ± 1.24a	18.33 ± 0.43bc	78.27 ± 2.12 cd	213.30 ± 2.91b	21.50 ± 0.49b	7.08 ± 0.04a	124.27 ± 3.50b
	T2	36.43 ± 0.46a	19.37 ± 0.50b	83.97 ± 2.99bc	212.00 ± 3.33bc	19.00 ± 0.76c	7.12 ± 0.03a	124.70 ± 6.15b
	T3	36.80 ± 0.81a	16.80 ± 0.67c	90.53 ± 1.92b	194.30 ± 8.79c	17.63 ± 0.43c	7.11 ± 0.03a	115.57 ± 6.99b
	T4	35.80 ± 1.80a	25.10 ± 0.93a	73.80 ± 2.62d	214.83 ± 4.33b	24.63 ± 1.18a	7.03 ± 0.03a	222.93 ± 6.01a
Day 90	CK	58.07 ± 1.79b	20.13 ± 0.68b	69.17 ± 1.75ab	206.70 ± 5.04a	12.53 ± 0.49c	7.32 ± 0.03b	96.83 ± 3.74c
	T1	67.10 ± 0.98a	21.47 ± 1.44b	60.63 ± 1.20b	164.50 ± 5.57bc	16.17 ± 0.45b	7.55 ± 0.04a	86.47 ± 5.80 cd
	T2	64.37 ± 1.58a	24.97 ± 0.87ab	73.53 ± 5.43a	174.90 ± 3.81b	16.43 ± 0.54b	7.40 ± 0.03b	230.23 ± 8.68a
	T3	64.60 ± 1.77a	22.70 ± 2.57b	75.63 ± 2.39a	152.67 ± 5.09c	15.00 ± 0.21b	7.41 ± 0.03b	70.77 ± 5.90d
	T4	58.17 ± 1.67b	27.83 ± 1.28a	62.20 ± 3.35b	149.13 ± 6.73c	21.97 ± 0.64a	7.18 ± 0.03c	129.10 ± 6.62b
Day 150	CK	36.37 ± 1.11a	20.77 ± 1.87b	52.57 ± 2.92ab	212.27 ± 3.15a	11.27 ± 0.26d	6.66 ± 0.04c	330.03 ± 10.32a
	T1	35.50 ± 0.40a	29.30 ± 2.08a	60.47 ± 3.50a	217.80 ± 5.23a	17.10 ± 0.15ab	6.89 ± 0.03b	196.90 ± 7.20bc
	T2	36.47 ± 0.58a	24.53 ± 2.23ab	45.47 ± 1.39b	215.53 ± 4.80a	16.00 ± 0.40c	7.03 ± 0.04a	212.70 ± 5.62b
	T3	34.33 ± 1.07a	24.27 ± 1.32ab	44.77 ± 2.07b	179.37 ± 1.62c	16.30 ± 0.47bc	6.99 ± 0.03a	184.77 ± 6.30c
	T4	35.27 ± 1.60a	23.03 ± 1.21b	44.27 ± 3.59b	197.53 ± 3.17b	17.83 ± 0.15a	7.07 ± 0.03a	181.90 ± 1.12c

Data depicts means ± SD of three biological replicates. Significant differences between treatments (*P* < 0.05) are illustrated by lowercase letters of a-d.

The content of AP was significantly lower in all OF treatments than in the CK at 30 days after treatment. The content of AP in all soil samples decreased over time. The content of soil AK was significantly lower in all OF treatments than in the CK at 30 d after treatment; the content of soil AK was the lowest in T3, and it was 18.49% lower in T3 than in the CK. The AK content was the lowest at 90 days after treatment in the CK, and the content of AK was 14.56 and 35.39% higher in T2 and T3 than in the CK, respectively. The AK content was 15.49 and 6.94% lower in T3 and T4 than in the CK at 150 days after treatment, and these differences were significant. Compared to chemical fertilizers, BOF treatments were more slow-release and helped to supplement the AK in the middle and late growth of *C. pilosula* (Yang et al., 2017). The content of OM significantly increased in the OF and BOF treatments during the three periods, and this was driven by the input of exogenous organic carbon (Table 1).

The soil pH was significantly higher in all BOF treatments than in the CK at 150 days after treatment. The excessive application of *CF* can result in the acidification of soil, which stems from the fact that ammonium sulfate and calcium carbonate make *CF* acidic (Kakihara and Ogura, 2022). The mixed application of cow manure and different OFs has been shown to increase soil pH to different degrees, which is consistent with the results of our study (Chen et al., 2023). Tobacco black shank is more common in acidic soil, and alterations in soil acidity can alter the abundance of the pathogens that cause tobacco black shank (Ma et al., 2023). Soil acidification also decreases the number of ammoniating bacteria and N-fixing bacteria in soil and the ammoniating and nitrification capabilities of soil microorganisms (Liu et al., 2023). The EC was significantly higher in T4 than in the other treatments at 30 days after treatment, and the EC was significantly higher in the CK than in the other four treatments at 150 days after treatment (Table 1).

### 3.3 Variation in the *α*- and *β*-diversity of soil microbial communities

**Figure 2:**
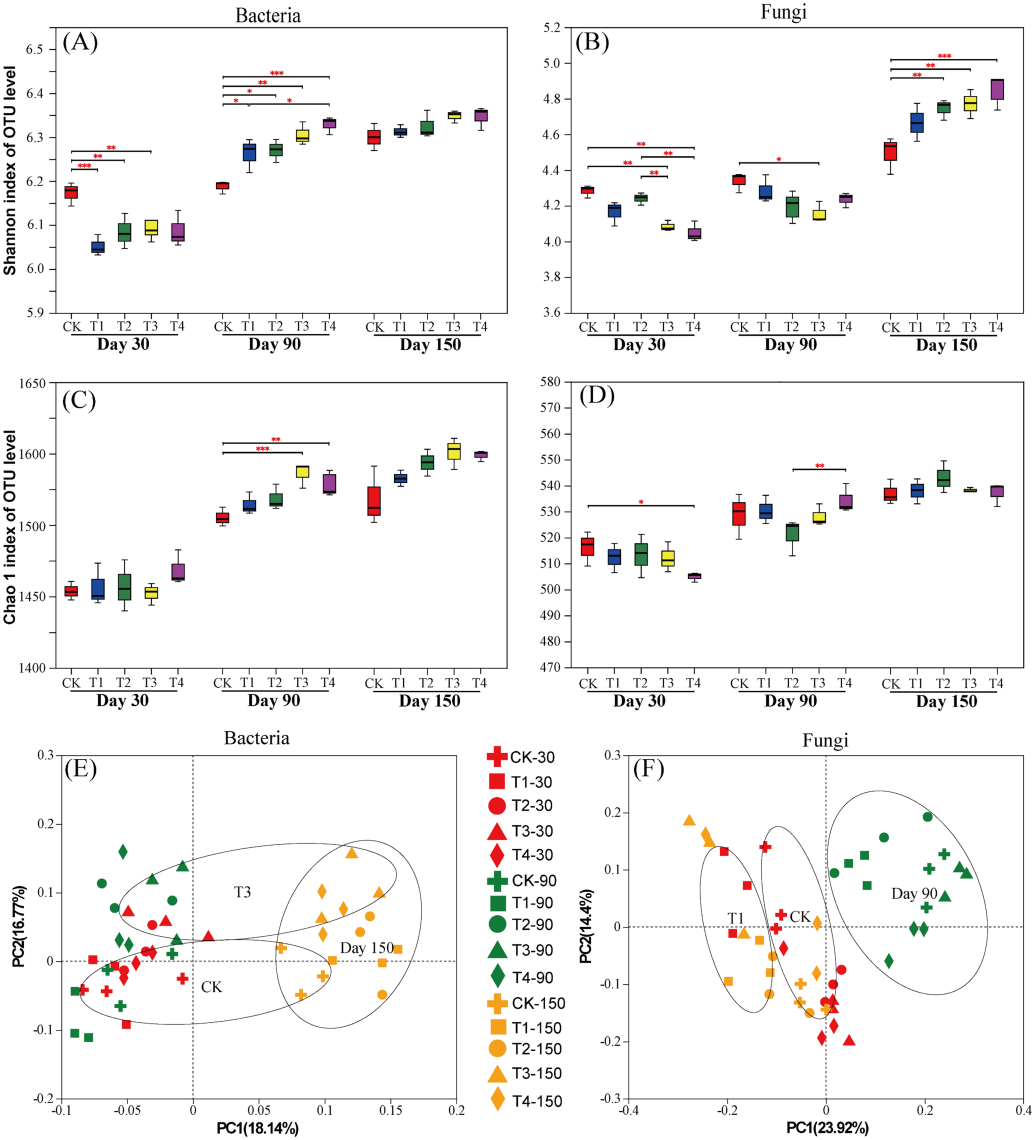
*α*- and *β*-diversity of rhizosphere soil microorganisms. (A) Shannon index of the soil bacterial community; (B) Shannon index of the soil fungal community; (C) Chao1 index of the soil bacterial community; (D) Chao1 index of the soil fungal community; (E) PCoA of the soil bacterial community; (F) PCoA of the soil fungal community.

The Shannon index of rhizosphere soil bacteria was significantly lower in T1, T2, and T3 than in the CK at 30 days after treatment. The Shannon index of bacteria was significantly higher in the OF and BOF treatments than in the CK at 90 days after treatment (Figure 2A). At 30 days after treatment, the Shannon index of soil fungi was the highest in the CK. At 150 days after treatment, the Shannon index of soil fungi was higher in T2, T3, and T4 than in the CK (Figure 2B). Thus, the diversity of soil microbes in BOF treatments was increased in the middle and later stages of the experiment. It may promote the stability and functional diversity of soil ecosystems (Sharma et al., 2022). The Chao1 index of soil bacteria was significantly higher in T3 and T4 than in the CK at 90 days after treatment (Figure 2C). The Chao1 index of soil fungi was significantly higher in the CK than in T4 at 30 days after treatment (Figure 2D).

The PCoA results indicated that the contribution of PC1 and PC2 to differences in the composition of species among treatments was 18.14%/16.77% (bacteria) and 23.92%/14.4% (fungi), respectively (Figures 2E,F). The community composition of soil bacteria at 150 days after treatment differed from that at 30 days and 90 days after treatment along PC1. The community composition of soil fungi at 90 days after treatment differed from that at 30 days and 150 days after treatment along PC1, which indicates that soil fungi were more vulnerable to the effects of BOFs in the middle of the experimental period compared to conventional fertilizers.

### 3.4 The driving factors affecting the emergence, disease prevention, yield, and quality of *C. pilosula*

**Figure 3:**
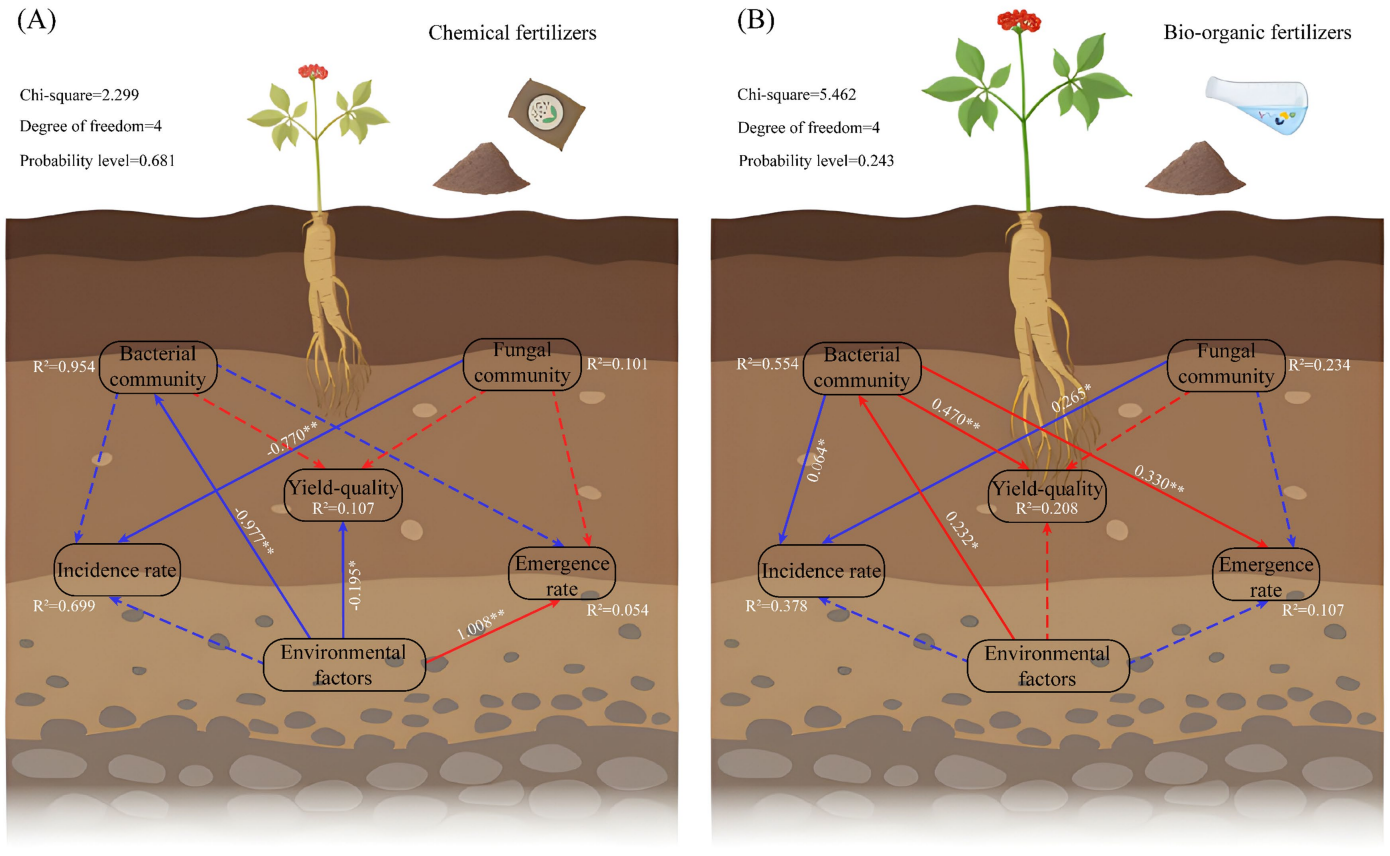
SEM of the effects of soil microbial community structure and environmental factors on the emergence rate, disease prevention, yield, and quality of *C. pilosula*. (A) SEM for the *CF* treatment; (B) SEM for the BOF treatment. Continuous and dashed arrows indicate significant and non-significant relationships, respectively. The numbers adjacent to the arrows indicate the path coefficients, and the red and blue arrows indicate negative and positive relationships, respectively. The *R*^2^ value indicates the proportion of the explained variance for each variable. The significance level was expressed as *p < 0.05, **p < 0.01, and ***p < 0.001. The total normalized effect of the structural equation model calculations is shown in the upper left. “Yield-quality” represents the yield, ash, and water content of *C. pilosula*; “Environmental factors” represents the soil physicochemical properties; “Bacterial community” represents the rhizosphere bacterial community structure; and “Fungal community” represents the rhizosphere fungal community structure.

To clarify the direct and indirect effects of soil environmental factors (NH_4_^+^-N, NO_3_^−^-N, AP, AK, OM, pH, and EC) and soil microorganisms on the emergence, disease prevention, yield, and quality of *C. pilosula*, we established a SEM for the *CF* (CK; Figure 3A) and BOF treatments (T2, T3, and T4; Figure 3B). Soil environmental properties accounted for the majority of the variation in the soil bacterial community (95.4%) in the *CF* treatment. Soil environmental properties were significantly negatively correlated with soil bacteria and yield quality and significantly positively correlated with emergence rate (path coefficient of 1.008). The soil fungal community was significantly negatively correlated with the incidence rate (path coefficient of −0.770).

Soil environmental properties explained the most variation in the soil bacteria community (55.4%) under BOF treatment. Soil environmental factors were significantly positively correlated with the soil bacterial community (path coefficient of 0.232). The soil bacterial and fungal communities were significantly positively correlated with incidence rate. The soil bacteria community showed a significant positive correlation with quality, yield, and emergence rate. Under the *CF* treatment system, soil environmental factors had a significant direct effect on the yield and quality of *C. pilosula*. However, under the BOF treatment, soil environmental factors directly affected the soil bacterial community, which directly drove the yield and quality of *C. pilosula*. CFs had a rapid effect and provided abundant nutrients; these nutrients are key for crops over short time intervals, given that CFs directly determine the yield and quality of crops. BOFs can promote N fixation, P solubilization, and K solubilization through the life processes of microbes; BOFs can also mediate reductions in molecular N in the atmosphere, the decomposition and destruction of mineral crystals through the production of various organic acids, the release of fixed P and K in the soil, and the full utilization of nutrients, which enhances the yield and quality of crops (Peng et al., 2022).

### 3.5 Differential microorganisms in soil bacterial communities

BOFs affect the incidence rate, quality, and yield of *C. pilosula* by influencing the soil bacterial community. Therefore, we focused on the changes in the soil bacterial community. Linear discriminant analysis effect size (LEFSe) was conducted to compare *CF* and BOF treatments (T2, T3, and T4) using data from the phylum to genus level, with an LDA threshold set at 2 (Supplementary Figure S1). In the soil bacterial community, p_unclassified_k_norank_d_Bacteria was significantly enriched in the BOF treatments, and no significant phylum was significantly enriched in the CK treatment. The orders Bryobacterales, Solibacterales, Cyanobacteriales, Acetobacterales, Reyranellales, and Sphingomonadaceae and the genera *Bryobacter*, *Reyranella*, and *Sphingomonas* were significantly enriched in the CK treatments. Solibacterales, Cyanobacteriales, and Sphingomonadaceae are all primitive microorganisms (Supplementary Figure S1). These archaea and bacteria inhabit swamps and volcanoes and prefer using simple nutrients to complete their life processes (Gao et al., 2018), which indicates that they would grow optimally with the direct nutrient supply provided by CFs but grow poorly with the indirect nutrient supply provided by BOFs. The orders Streptosporangiales and Rubrobacterales, and the genera *Microlunatus* and *Rubrobacter* were significantly enriched in BOF treatment. Many genera in Streptosporangiales produce broad-spectrum antibiotics, including *Streptospora rosetta* and *Streptospora virescini*. They can produce polymycin and sporomycin, respectively, and inhibit most plant pathogens. Streptosporangiales may be the key differentiating bacteria in the prevention of the root rot of *C. pilosula*. SG1 strain of Streptosporangiales showed significant positive results in its control effect on cucumber fusarium wilt and its growth promotion effect on durum wheat. Among them, SG1 can reduce the disease index of cucumber wilt from 77.8 to 16% and significantly increase wheat growth and yield, thus having a wide application prospect (Nassira et al., 2018). *Microlunatus* and *Rubrobacter* were isolated from Marine ascidians with obvious antibacterial and anti-inflammatory activities. The former has anti-*Candida albicans* activity, and the ethyl acetate extract of the latter has good cytotoxic activity (Yuan et al., 2014; Chen et al., 2018). They may play a key role in the prevention of the root rot of *C. pilosula*.

### 3.6 Co-occurrence network analysis of soil bacteria community

**Figure 4:**
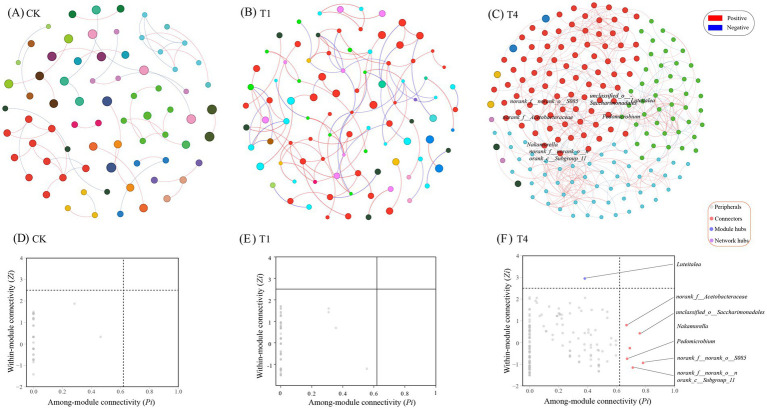
Analysis of the co-occurrence network of soil bacteria community induced by CK, OF, and BOF. (A) Co-occurrence network of soil bacterial community after CK (chemical fertilizer). (B) Co-occurrence network of soil bacterial community after organic fertilizer (OF, T1). (C) Co-occurrence network of soil bacterial community after BOF (T4). (D) Distributions of keystone taxa according to the topological properties of CK. (E) Distributions of keystone taxa according to the topological properties of OF. (F) Distributions of keystone taxa according to the topological properties of BOF (T4). The colors of the different nodes indicate different microbial genera, with red edges indicating positive correlations between two nodes and blue edges indicating negative correlations.

Co-occurrence networks for the bacterial communities in the *CF* (CK), OF (T1), and BOF (T4) treatments were constructed to clarify the effects of these fertilizers on the interactions between soil microorganisms. In the soil bacterial co-occurrence network, the CK network included 73 nodes and 56 edges; the OF network included 95 nodes and 102 edges; and the BOF (T4) network included 193 nodes and 506 edges (Supplementary Table S2). The number of nodes and connections in the co-occurrence network of bacteria increased following the application of OFs and BOFs (T4), and the number of nodes and connections was the highest in the BOF (T4) treatment, suggesting that the soil microbial network was more complex and interactions were stronger in the BOF treatment than in the *CF*/OF treatment. Nodes were classified by their within-module connectivity (*Zi*) and among-module connectivity (*Pi*), and their roles in the network were deduced. In the bacterial network, all nodes of CK and OF were identified as peripherals, with no critical nodes. Under the treatment of BOF, *Luteitalea*, *Nakamurella*, *Pedomicrobium*, *norank_f__Acetobacteraceae*, *unclassified_o__Saccharimonadales*, *norank_f__norank_o__S085*, and *norank_f__norank_o__norank_c__Subgroup_11* were identified as the key class groups (Figure 4). Of these key groups, one is a Module hub, which is *Luteitalea*. The remaining six groups are connectors, which are highly connected nodes between modules and can combine different modules to achieve network functions; therefore, the realization of BOF to reduce the incidence of *C. pilosula* and improve the emergence rate, quality, and yield of *C. pilosula* may require the joint action of different modules to complete.

*Luteitalea*, as a phosphorus-solving bacterium in soil, contains *ppgC* gene and can produce organic acid and reduce calcium-bound phosphorus, thereby improving the utilization efficiency of phosphorus by plants (Ding et al., 2022). *Nakamurella* is also sensitive to the antibiotic ciprofloxacin and can be used as a marker of environmental ecological health (Kim et al., 2020). *Pedomicrobium* has been shown to degrade some of the microplastics in soil and repair soil pollution (Rong et al., 2021). Current studies believe that the complexity of the microbial co-occurrence network is a key factor reflecting the interrelation and coexistence of microbes in an ecological environment (Kishore et al., 2023).

### 3.7 Microbial function analysis of driving emergence, disease prevention, yield, and quality change of *C. pilosula* at different times

**Figure 5:**
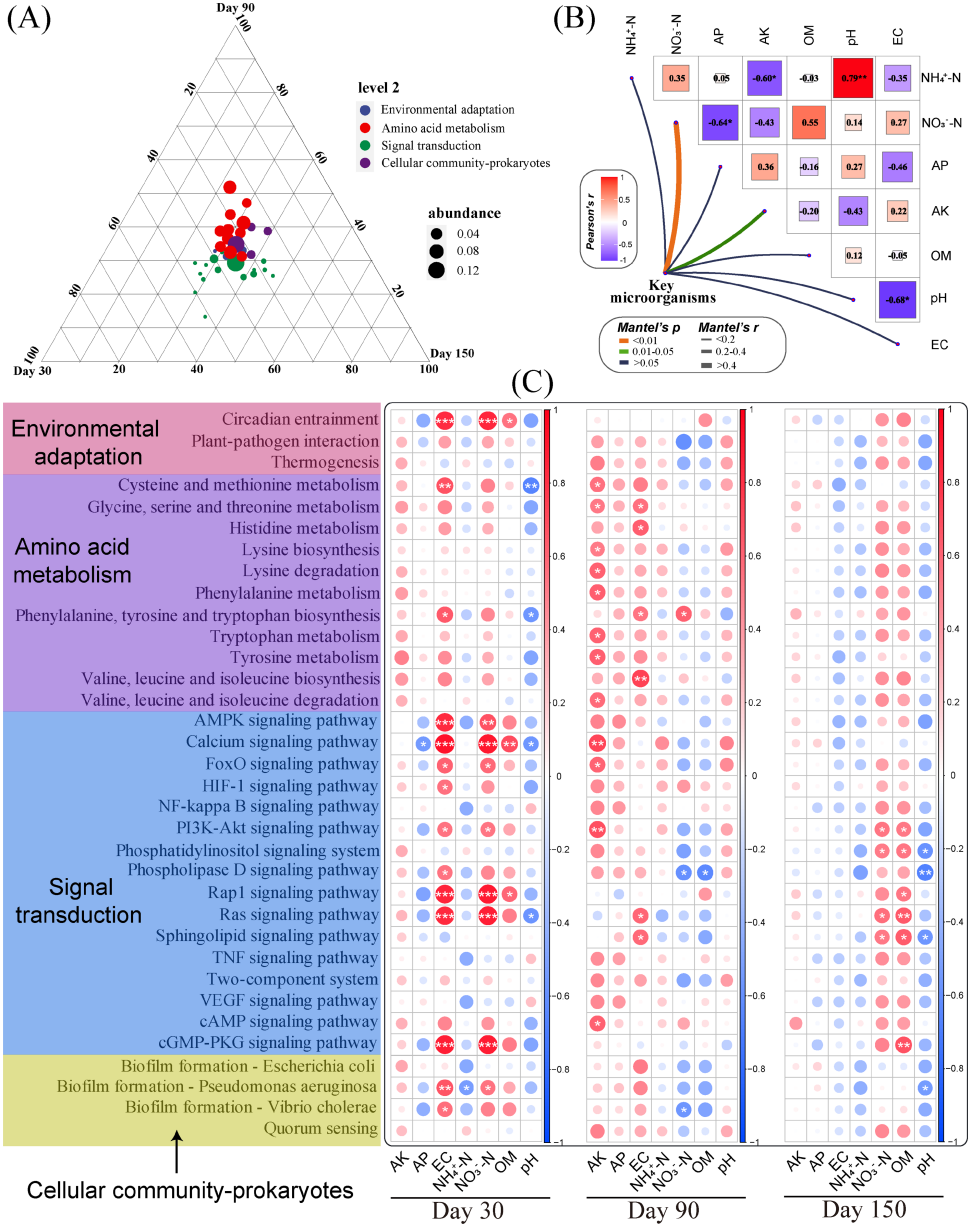
Soil microbial function analysis at different times. (A) Ternary phase analysis of the relative abundance of KEGG level 2 functional genes; (B) Mantel detection of key microorganisms and soil environmental factors; (C) Correlation analysis of the relative abundance of KEGG level 3 functional genes and soil environmental factors in three periods. The significance level was expressed as *p < 0.05, **p < 0.01, and ***p < 0.001.

The differential microorganisms obtained from the LEFSe analysis and the key microbial class identified in network analysis after BOF treatment may be effective microorganisms for reducing root rot of *C. pilosula* and improving yield quality, which we defined as key microorganisms. The relative abundance matrix of their OTU level and the physicochemical properties of soil were used for the Mantel detection (Figure 5B) to characterize the correlation between key microorganisms and soil environmental factors. The results showed that key microorganisms were strongly correlated with NO_3_^−^-N (*p* < 0.01) and AK (*p* < 0.05), but not strongly correlated with other soil environmental factors. At the same time, there was a significant positive correlation between soil pH and NH_4_^+^-N (*r* = 0.79) and a significant negative correlation between pH and EC (*r* = −0.68), AK and NH_4_^+^-N (*r* = −0.60), AP and NO_3_^−^-N (*r* = −0.64).

In addition, we constructed a ternary plot of functional genes at level 3 over three time points (Figure 5A) and a correlation analysis between the relative abundance of functional genes and environmental factors (Figure 5C) to explore how different times and functions affect the yield and quality of *C. pilosula*. The results showed that the signal transduction was closer to day 30 and day 150, and the amino acid metabolism was closer to day 90. The results of correlation analysis showed that the function of key microorganisms was positively correlated with EC on day 30, AK on day 90, and OM on day 150. After that, we selected 10 functional genes with strong correlation at level 3 (5 on day 30, 3 on day 90, and 2 on day 150) for further analysis, and the results showed that, on day 30, the relative abundance of calcium signaling pathway, Rap1 signaling pathway, Ras signaling pathway, and cGMP-PKG signaling pathway in T4 was the highest. Both were significantly higher than the CK treatment (Supplementary Figure S2). During the initial application of the BOF treatment (especially bacteria), a large number of *Bacillus* as “invaders” broke the balance of the soil ecosystem, and the soil native bacterial community maintained the balance of the ecosystem by strengthening signal transduction. Calcium, Rap1, Ras, Cgmp-PKG, and other signal pathways are important intracellular signal transmission systems. They are involved in conducting many hormone signaling pathways (inducing or inhibiting the synthesis of hormones within plants to affect crop yield and quality) (Zhang et al., 2022), metabolite signaling pathways (secreting enzymes directly affect the nutrient absorption and metabolic processes of crops) (Wang et al., 2023), and immune system signaling pathways (regulating the plant immune system to affect crop yield and quality) (Joseph et al., 2019). On day 90, glycine, serine, and threonine metabolism in T2 and T3 were significantly higher than those in CK, and valine, leucine, and isoleucine biosynthesis in T2 were significantly higher than those in CK. The biosynthesis of these amino acids is one of the functions of metabolites and plants to drive the change of diseased soil, and their metabolism may play a certain role in the weakening of the root rot of *C. pilosula* (Li et al., 2018; Wen et al., 2022). On day 150, the sphingolipid signaling pathway in T4 was significantly higher than that in CK. Sphingolipids, as one of the important components of the cell membrane, participate in various physiological processes (Sánchez-Rangel et al., 2015), and play an important role in the stability of the rhizosphere bacterial community at the later stage of the experiment.

Based on the species and functional relative abundance of the samples, the relationship between key microorganisms’ abundance and functional abundance was analyzed to explore the specific microorganisms that performed major functions during the three periods (Supplementary Figure S3). The results show that *Nakamurell* contributed most to the calcium signaling pathway, phenylalanine, tyrosine, and tryptophan biosynthesis, Rap1 signaling pathway, cGMP-PKG signaling pathway, and Ras signaling pathway, which were 54.03, 51.17, 50.62, 50.92, and 26.03%, respectively. *Pedomicrobium* contributed most to glycine, serine, and threonine metabolism, phosphatidylinositol signaling system, and sphingolipid signaling pathway, which were 43.85, 33.74, and 27.19%, respectively. *Microlunatus* contributed most to biofilm formation—*Pseudomonas aeruginosa* (32.78%). *Rubrobacter* contributed most to the biosynthesis of valine, leucine, and isoleucine (33.23%).

## 4 Conclusion

In this study, the yield and quality of *C. pilosula* were higher, and the disease incidence was lower in the BOF treatment than in the *CF* treatment. These findings indicate that soil ecology was enhanced by the application of BOFs. The addition of BOFs promoted the diversity and richness of rhizosphere bacteria in the middle growth stage of *C. pilosula*. The SEM showed that the rhizosphere bacterial community directly drove the yield and quality of *C. pilosula* after BOF application, while the chemical fertilizer increased the yield and quality by influencing soil environmental factors. LEFSe and network analysis showed that *Microlunatus*, *Rubrobacter*, *Luteitalea*, *Nakamurella*, and *Pedomicrobium* as key microorganisms, may play a key role in improving the soil of *C. pilosula*. At the same time, the microbial co-occurrence network revealed stronger interactions between soil microorganisms after the BOF treatment, which are key to soil biodiversity and ecosystem stability. Key microorganisms increase the signal transduction and amino acid metabolism functions of the rhizosphere bacterial community in the early and middle growth period of *C. pilosula*. *Nakamurell* and *Pedomicrobium* made a major contribution to the change in microbial function. These results concluded that BOFs and CFs have different mechanisms for improving the yield and quality of Chinese herbal medicine. BOFs are more beneficial to soil ecological health.

## Data Availability

The datasets presented in this study can be found in online repositories. The names of the repository/repositories and accession number(s) can be found in the article/Supplementary material.
