# Supplementary material for: The structure and function of rhizosphere bacterial communities: impact of chemical vs. bio-organic fertilizers on root disease, quality, and yield of Codonopsis pilosula
Source: Front Microbiol. 2024 Oct 21;15:1484727. doi: 10.3389/fmicb.2024.1484727 (PMC11532114; doi:10.3389/fmicb.2024.1484727)
Supplement: Supplementary file 1 [file Data_Sheet_1.pdf]

Supplementary materials

Title: The structure and function of rhizosphere bacterial community contributed to the difference between bio-organic fertilizer and chemical fertilizer in root disease, quality and yield of *Codonopsis pilosula*

Authors: Bin Huang[1,†], Yuxuan Chen[1,†], Yi Cao[2*], Dongyang Liu[4], Hua Fang[5], Changchun Zhou[5], Dong Wang[3*], Jie Wang[1*]

[1]Pest Integrated Management Key Laboratory of China Tobacco, Tobacco Research Institute of Chinese Academy of Agricultural Sciences, Qingdao 266101, China

[2]Guizhou Academy of Tobacco Science, Guiyang 550000, China

[3]Department of Vector Biology and Control, Jinan Center for Disease Control and Prevention, Jinan, Shandong 250021, P.R. China

[4]Institute and Enterprise Joint Creation of Tobacco Technology Center, Sichuan Provincial Tobacco Company Liangshanzhou Company, Liangshanzhou 615000, China

[5]Shandong Hezhong Kangyuan Biotechnology Co., LTD, Zibo, Shandong 255000, China

[*]Correspondence to: Jie Wang, No. 11, Keyuanjingsi Road, Laoshan District, Qingdao City, Shandong Province, Qingdao 266101, China; Email: wangjie@caas.cn. Phone: +8610-88702117. Dong Wang, No. 2, Weiliu Road, Jinan, Shandong 250021, P.R. China; E-mail: 9830129@163.com; Phone: 86-0531-81278980.

[1] These authors contributed equally to this paper.

Table S1. Effects of CF, OF and BOF on the emergence rate, disease index, yield and quality of *Codonopsis pilosula*

Treatment	emergence rate (%)	disease index	water content (%)	ash content (%)	yield (kg/ha)
CK	67.62±0.63c	11.11±0.28a	11.47±0.18a	4.87±0.06a	6466.35±96.3c
T1	71.19±0.24bc	6.52±0.28b	10.75±0.16b	4.04±0.05b	7312.2±78.9b
T2	72.38±1.33b	3.33±0.28c	10.68±0.07bc	4.05±0.03b	7532.1±82.8b
T3	81.90±1.45a	2.88±0.55c	10.43±0.07bc	4.03±0.09b	8443.2±91.65a
T4	81.19±1.67a	2.60±0.28c	10.31±0.06c	4.00±0.05b	8642.4±63.9a

Table S2. Topological characteristics of microbial co-occurrence networks of chemical fertilizer (CK), organic fertilizer (OF) and bio-organic fertilizer (BOF)

Network	Topological properties	Bacteria community		
		CK	OF	BOF
Fruchterman Reingold Networks	Total nodes	73	95	193
	Total links	56	102	506
	Average degree	1.534	2.842	5.244
	Average clustering coefficient	0.400	0.406	0.415
	Network diameter	5	7	11
	Network density	0.021	0.023	0.027
	Modularity	0.902	0.895	0.567
	Average path length	1.812	2.385	4.37

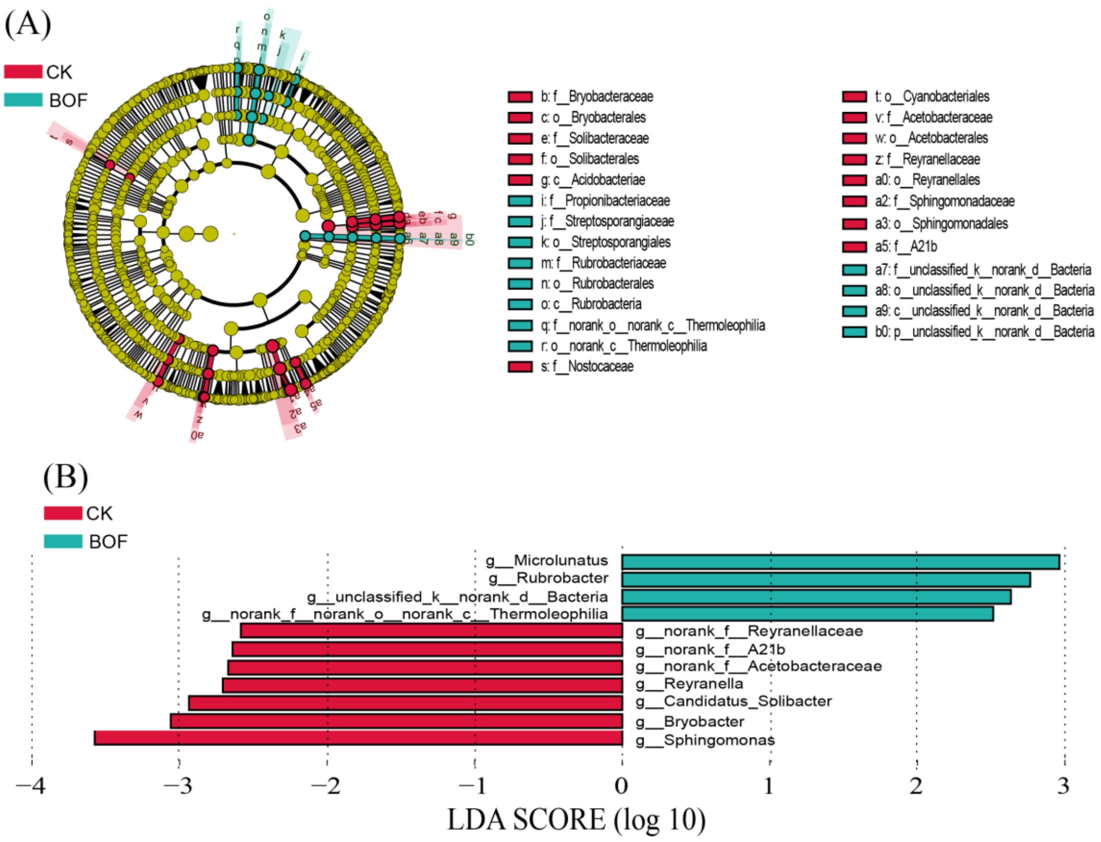

Fig.S1. LEFSe of soil microorganisms in the CK and BOF.

(A) Phylogeny of bacterial lineages from the phylum level to the family level in soil treated with CK and BOF (LDA$\geqslant$ 2.0); (B): Soil bacterial communities at the genus level with LDA score greater than 2 after CK and BOF.

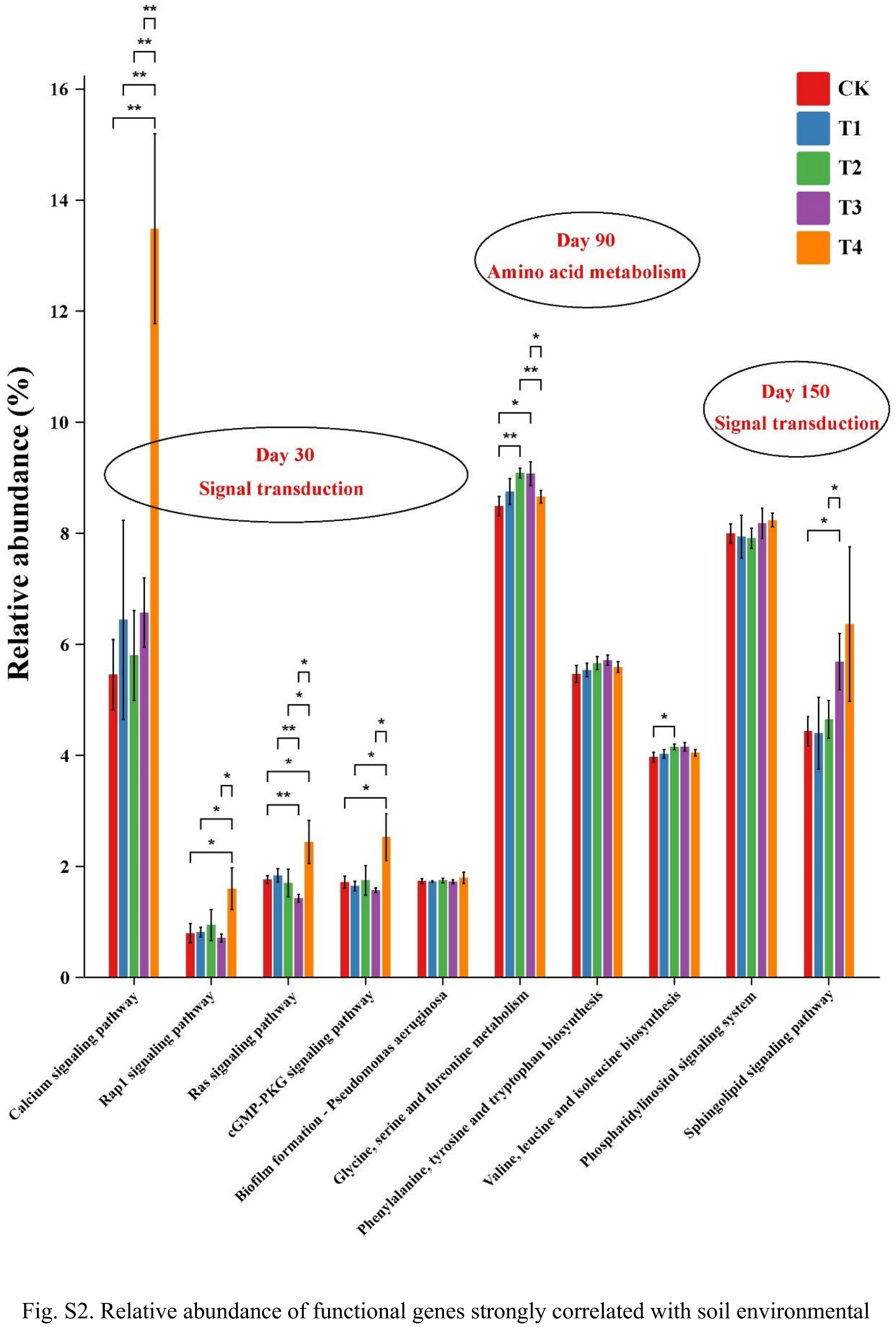

Fig. S2. Relative abundance of functional genes strongly correlated with soil environmental factors of three periods in different treatments.

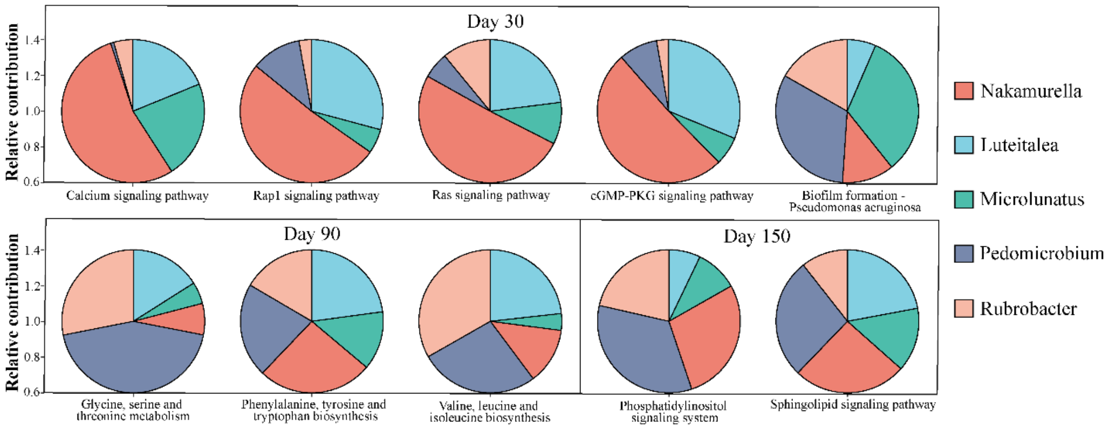

Figure S3. The contribution of effector microorganisms to different functions of soil bacteria in three periods.
